# Improved Forceps, &c.

**Published:** 1857-01

**Authors:** Samuel Maclean


### ARTICLE XV.

### *Improved Forceps, &c.* By SAMUEL MACLEAN, ESQ.

AT a meeting of the *Royal Medical and Chirurgical Society of London*, a paper was read by Samuel Maclean, Esq., late of Dublin, but now of Brook street, Grovenor Square, London "*on the systematic removal of the four permanent first molars at an early period, in a large majority of cases, when incipient caries is present.*"

This mode of treatment is not new. It was recommended by Mr. Fox in 1803, and is supported by the author, although it has not received general support. The advantages are:

1. The prevention and correction of the simpler forms of irregularities in the easiest and most desirable way, in a great majority of cases, without the aid of mechanical means; in all, in such a manner as least to disfigure the appearance of the mouth.

2. The promotion of a healthier state amongst the remaining teeth; the prevention (probably) of caries, certainly an increase in the facility of treating it.

3. The prevention of the distressing, in some cases even very serious, symptoms which frequently accompany the development of the wisdom teeth in corroded arches, and a material diminution in the chance of the formation of sinuses in after-life.

These statements were supported by cases, illustrated by numerous accurately-executed casts. The author also exhibited forceps of a novel construction, by which he was enabled to remove carious teeth. Each instrument consisted of a pair of narrow forceps sliding over one another, so as to be able to grasp the tooth in two different levels.

Dr. Copland remarked that the plan proposed by Mr. Maclean was one followed in agriculture. When plants were too crowded they were thinned.

Mr. John Thomson inquired whether the author of the paper recommended the removal of the deciduous teeth one or two years before the appearance of the permanent set? The author had prefixed an *a** to the name of Mr. Robinson in his paper—a gentleman surely well enough known not to require to be so styled. He (Mr. Thomson) would refer to a case similar to the one recorded by Mr. Robinson. In that instance the deciduous teeth had been removed two years before the permanent ones appeared, and the result had been that these were all zigzag. He mentioned another case also, of a young lady now thirteen years of age, who was born with two incisor teeth of the lower jaw. The family surgeon removed them soon after, and this young lady has now every one of her permanent teeth with the exception of these two and the dentes sapientiæ. Had the removal of the teeth, as stated, the effect of keeping up the deficiency?

Mr. Maclean called attention to the casts of Mr. Nasmyth, which represented the mouth of a lady who never had more than sixteen permanent teeth, yet the circle of the arch remained perfect, and did not adapt itself to the number of teeth in it. He considered that Mr. Robinson could not support his censure of the dentist referred to in his work, on the memory of the parent after a lapse of seven years. In his (Mr. Maclean's) paper he mentioned that the arch was a rabbit malformed one. In answer to Mr. Thomson, he said that he never removed primary teeth *except in cases where an irregularity was established* by them, and that the fangs of the primary were not always removed by anticipative absorption.

Dr. Snow said that the operation of removing four molar teeth in a child was one requiring the use of chloroform, and there was hardly any operation in which the benefits of this agent were better shown. He had exhibited it in a great number of such cases, and the results had always been very satisfactory.

*This referred to the manner in which the author of the paper, when combating certain views advanced by Mr. Robinson, spoke of that gentleman.—Rep. L.

Mr. Vasey said that the author, in referring to the admirable statistics of Mr. Tomes, had expressed his surprise that, out of more than a thousand permanent first molar teeth extracted, one only was for the purpose of regulation, and had entirely overlooked the fact, that those persons who apply at such an institution as that mentioned by Mr. Tomes belong to that class who care nothing whatever for the regular arrangement of the teeth—in fact, they apply with the teeth utterly decayed, and requiring immediate extraction. The profession, he believed, well understood those statistics, which admirably showed, as far as they went, the relative liability of the different teeth to decay. It was a general rule that when we required to extract a tooth for the purpose of regulating over-crowding, we invariably extracted the first permanent molar if decayed. We had to consider each case by itself, how the teeth closed upon each other, &c.; and when the teeth were all sound it was sometimes better to extract the posterior bicuspid next to the anterior one. With respect to the forceps exhibited, they evidently were of little use, except to crush the teeth. The simple principle on which forceps should be constructed, and which he thought was well understood by every dentist, seemed to be entirely lost sight of in the present instrument. A tooth that was much decayed might be considered like a short hollow cylinder or tube, and if grasped by four points, as it would be by these forceps, it must inevitably be crushed to pieces. The principle of one tube fitting on to another seemed to be overlooked. A mere shell of a tooth, when grasped by forceps fitted on such a principle, would bear an enormous amount of pressure. The author surely could not be aware of what could be done with proper forceps, such as those so admirably adapted by Mr. Errard. He (Mr. Vasey) had teeth in his collection, completely hollowed out nearly to the division of the fangs, that he had easily extracted with Errard's forceps.—*London Lancet.*

